# Integrating chemokine signatures and multi-omic biomarkers to predict immunotherapy response in non-small cell lung cancer: a comprehensive narrative review

**DOI:** 10.3389/fonc.2026.1830731

**Published:** 2026-07-02

**Authors:** Luis Cabezón-Gutiérrez, Magda Palka-Kotlowska, Sara Custodio-Cabello, Adriana Carolina Rosero-Rodriguez, Beatriz Chacón-Ovejero

**Affiliations:** 1 Department of Medical Oncology, Hospital Universitario de Torrejón, Torrejón de Ardoz, Spain; 2 Facultad de Medicina, Universidad Francisco de Vitoria, Pozuelo de Alarcón, Spain; 3 Department of Pharmacy and Nutrition, Faculty of Biomedical and Health Sciences, Universidad Europea de Madrid, Madrid, Spain

**Keywords:** biomarkers, chemokines, immunotherapy, multi-omics, NSCLC, precision oncology, predictive biomarkers, tumor microenvironment

## Abstract

Non-small cell lung cancer (NSCLC) is the leading cause of cancer-related mortality worldwide. While immune checkpoint inhibitors (ICIs) targeting PD-1/PD-L1 and CTLA-4 have revolutionized the therapeutic landscape, only 20–30% of unselected patients achieve durable clinical benefits. Given the imperfect predictive value of traditional markers, such as PD-L1 expression and tumor mutational burden, there is an urgent need for multidimensional biomarkers to guide personalized immunotherapy. This review evaluates emerging predictive tools, with a specific focus on chemokine signatures and multi-omic (genomic, transcriptomic, proteomic, and metabolomic) biomarkers, including integrative models. By examining the biological rationale linking tumor microenvironment chemokine networks to antitumor immunity, we discuss recent advances in profiling that enable comprehensive predictive signatures. A comprehensive narrative literature search of PubMed and EMBASE (2015–2026) was performed to identify relevant peer-reviewed studies, clinical trials, and computational analyses. Evidence suggests that integrating chemokine profiles with multi-omic data holds significant promise for improving patient selection. Multidimensional models incorporating tumor genomics and immune microenvironment features are likely to outperform single-analyte tests in identifying ICI responders. Despite ongoing challenges, such as tumor heterogeneity, assay standardization, and data integration complexity, the development of liquid biopsies and advanced machine learning models offers a path toward robust, clinically applicable predictive nomograms, which are expected to refine immunotherapy decision-making and significantly improve clinical outcomes for patients with NSCLC.

## Highlights

- Immune checkpoint inhibitors have transformed the treatment of advanced NSCLC; however, only approximately 20–30% of patients achieve durable responses. This underscores the urgent need for better predictive biomarkers beyond PD-L1 expression and tumor mutational burden (TMB) to identify patients who are likely to benefit from immunotherapy more accurately.
- Tumor-derived chemokines are key orchestrators of the tumor immune microenvironment and serve as potential biomarkers for immunotherapy response. Pro-inflammatory T_h1_ -type chemokines (such as CXCL9, CXCL10, and CXCL13) are associated with immune “hot” tumors and favorable responses to checkpoint inhibitors, whereas high levels of immunosuppressive chemokines (e.g., IL-8 and CCL2) correlate with T-cell exclusion and resistance to therapy.
- Multi-omic integration (combining genomic, transcriptomic, proteomic, and metabolomic data) yields robust predictive models for immunotherapy outcomes in patients with NSCLC. By integrating tumor genetic features (including TMB and specific mutations), immune gene expression signatures, and circulating biomarkers, these composite approaches outperform single biomarkers in distinguishing responders from non-responders.
- Specific genetic alterations and immune features of NSCLC tumors influence the efficacy of checkpoint inhibitors. For example, tumors harboring co-mutations in *STK11/LKB1* or *KEAP1* often exhibit “cold” immune phenotypes and poor responses to PD-1/PD-L1 inhibitors, even when the TMB is high. In contrast, tumors characterized by high neoantigen loads, active interferon-γ pathways, or the presence of tertiary lymphoid structures tend to be more immunogenic and respond better to immunotherapy.
- Emerging composite biomarkers and machine learning models integrate diverse data (tumor DNA mutations, RNA expression profiles, immune cell metrics, and serum factors) to predict immunotherapy benefits with greater accuracy. These integrative risk scores have demonstrated improved performance over PD-L1 or TMB alone, marking a step toward personalized immunotherapy selection, although they require prospective validation in clinical trials.
- This review highlights that combining chemokine profiling with multi-omic biomarkers can refine patient selection for immunotherapy in NSCLC. Future approaches may include real-time monitoring of tumor and immune dynamics through liquid biopsies and targeting immunosuppressive chemokine pathways, alongside checkpoint blockade. These strategies aim to increase response rates and tailor treatment plans to each patient’s immunological tumor profile.

## 1 Introduction

Lung cancer remains the leading cause of cancer-related mortality worldwide, with non-small cell lung cancer (NSCLC) accounting for approximately 85% of cases (1). The development of immune checkpoint inhibitor (ICI) therapies, particularly antibodies targeting programmed cell death-1 (PD-1), programmed death-ligand 1 (PD-L1), and cytotoxic T-lymphocyte-associated protein 4 (CTLA-4), has transformed the treatment landscape of advanced NSCLC (2). These immunotherapies can induce durable tumor regression and prolonged survival in a subset of patients, even with historically poor prognosis disease (3). Consequently, agents such as pembrolizumab, nivolumab, and atezolizumab have become the standard of care for advanced NSCLC, either as monotherapy or in combination with chemotherapy (4). However, only ~20–30% of unselected NSCLC patients experience a significant and lasting response to single-agent ICIs, whereas many others derive no benefit (5). This inherent variability underscores the imperative for robust predictive biomarkers to stratify patients who will derive maximal clinical benefit from immunotherapy versus those who require alternative therapeutic interventions (6).

Currently approved biomarkers for NSCLC immunotherapy include tumor PD-L1 expression (by immunohistochemistry) and tumor mutational burden (TMB). High tumor PD-L1 expression (e.g., ≥50% tumor cell staining) correlates with higher response rates to first-line anti–PD-1 therapy and has been used since 2016 to select patients for pembrolizumab monotherapy (7). Similarly, high TMB (e.g., ≥10 mutations per megabase) has been associated with improved ICI response in multiple studies, presumably because tumors with more mutations generate more neoantigens to stimulate T cells (8, 9). In 2020, the U.S. FDA approved pembrolizumab for solid tumors with high TMB, reflecting the potential of this biomarker in immunotherapy selection (10).

Despite these clinical milestones, both PD-L1 and TMB exhibit important predictive limitations. PD-L1 expression is characterized by significant intratumoral heterogeneity and spatiotemporal fluctuations, which may render single-point biopsy assessments insufficient. Similarly, TMB estimation requires substantial tumor cellularity, entails significant analytical costs, and exhibits inter-platform variability, such that the establishment of a robust universal threshold remains a subject of ongoing debate (11, 12). Furthermore, neither marker is perfectly predictive in isolation: some patients with low or negative PD-L1 expression still respond to ICIs, whereas many with high PD-L1 expression do not, and not all TMB-high tumors respond to therapy (3, 5). These observations have driven intense interest in novel, noninvasive, and multi-parametric biomarkers capable of capturing the dynamic complexity of antitumor immune responses. Among the most promising candidates are circulating immune markers (particularly chemokines and cytokines) and composite multi-omic signatures that integrate genomic mutations, gene expression profiles, and protein levels into robust predictive models (7, 8).

Chemokines play fundamental roles in tumor immunology, influencing T-cell infiltration, immune cell differentiation, and the balance of pro- and anti-tumor factors in the tumor microenvironment (TME) (13). High-throughput “omics” technologies and advanced computational approaches have enabled the discovery of complex biomarker combinations that may surpass the predictive power of individual analytes (14, 15). In this comprehensive review, we summarize the current state of knowledge regarding chemokine-based biomarkers and multi-omic predictors of immunotherapy response in patients with NSCLC. We discuss the biological rationale linking chemokine networks to antitumor immunity, highlight recent studies identifying specific chemokine signatures and integrated multi-omic models associated with ICI outcomes, and examine the challenges in translating these findings to clinical practice. We also consider future directions, including the integration of chemokine profiling with multi-omic liquid biopsy biomarkers and advanced computational methods, to improve patient selection and guide personalized immunotherapy in NSCLC.

## 2 Materials and methods

We performed a comprehensive narrative literature search to identify publications on biomarkers and predictors of immunotherapy response in NSCLC, with an emphasis on chemokine-related markers and multi-omics approaches. The search covered literature published from January 2015 to January 2026. The primary databases queried were PubMed and Embase. Search terms included combinations of “non-small cell lung cancer,” “immunotherapy,” “checkpoint inhibitor,” “PD-1,” “PD-L1,” “biomarker,” “predictors,” “chemokine,” “cytokine,” “gene expression,” “multi-omics,” “genomic,” “proteomic,” “metabolomic” and related terms. We also specifically searched for known biomarkers such as “PD-L1,” “tumor mutational burden,” “TMB,” “chemokine signatures,” “CXCL,” “CCL,” “IL-8,” “CXCL9,” “CXCL10,” “CXCL13,” “STK11,” “KEAP1,” “gene expression profile immunotherapy,” “T cell inflamed GEP” and “multi-omic immunotherapy prediction.” More than 300 articles were screened based on their titles and abstracts. Following the initial search, titles and abstracts of all identified records were screened independently by two reviewers, with discrepancies resolved by consensus. After full-text review of potentially eligible articles, a total of 109 references were ultimately included in the final narrative synthesis. We included peer-reviewed English-language articles reporting clinical trials, retrospective studies, meta-analyses, and translational or computational research on NSCLC immunotherapy biomarkers. Priority was given to studies with larger patient cohorts (particularly ≥50 patients) and those published since 2018 to capture recent advances. The exclusion criteria were case reports, non-English papers, and studies focusing on biomarkers of therapeutic modalities other than immune checkpoint blockade. The identified literature was reviewed and organized into thematic categories: (1) chemokines and the NSCLC tumor immune microenvironment (biological rationale and specific chemokine biomarkers), (2) tumor-intrinsic genomic and transcriptomic predictors, (3) host and circulating biomarkers (e.g., blood-based immune and proteomic markers), (4) multimodal integrative predictive models, and (5) challenges and future directions. We extracted and tabulated the key findings of chemokine signatures and multi-omic biomarker studies. This review synthesizes evidence from clinical and translational studies to provide a comprehensive overview of the current and emerging immunotherapy biomarkers in NSCLC. Because this is a narrative rather than a systematic review, no formal GRADE or ROBINS-I quality assessment was applied. Study robustness was, however, evaluated informally based on the following criteria: sample size, availability of an independent validation cohort, the study design (prospective trial versus retrospective cohort), and whether findings had been replicated in more than one dataset. Studies were distinguished in the narrative and in the tables according to whether the supporting evidence derives from (i) retrospective exploratory analyses, (ii) translational substudies of clinical trials without pre-specified biomarker endpoints, (iii) externally validated retrospective models, or (iv) prospectively evaluated biomarkers. Where the same patient dataset or biomarker was reported across overlapping publications, the most complete or most recently published analysis was prioritized, and overlapping datasets were noted to avoid double-counting. Findings highlighted in the main text were selected because they represent the most robust, biologically coherent, or clinically advanced evidence within each topic area; studies with only single-cohort, unreplicated findings are noted as such and cited primarily to illustrate emerging trends rather than to make strong clinical recommendations.

## 3 Chemokine signatures and the tumor immune microenvironment in NSCLC

Chemokines (chemotactic cytokines) form a network of >50 small secreted proteins that direct the migration and positioning of immune cells via interactions with G protein–coupled chemokine receptors (for example CCR1–10, CXCR1–6) (13). In NSCLC and other cancers, chemokine pathways are often dysregulated, profoundly affecting tumor development and response to therapy (16). Chemokines can have dual and context-dependent roles, either promoting effective antitumor immunity (by recruiting effector leukocytes) or facilitating immune evasion and tumor progression (by attracting immunosuppressive cells and fostering pro-tumor inflammation) (17). This duality means that distinct chemokine signatures (defined by the pattern of chemokine and chemokine receptor expression in the tumor and circulation) can serve as biomarkers of an immune-inflamed (“hot”) versus immune-suppressed (“cold”) TME (18). Given that the presence or absence of a pre-existing anti-tumor immune infiltrate within the TME is a pivotal determinant of ICI efficacy, chemokine profiling provides a comprehensive ‘window’ into the patient’s ‘immune contexture.’ Consequently, these signaling molecules have emerged as high-potential predictive biomarkers for tailoring immunotherapy outcomes (19). **Table 1** summarizes the selected chemokine-related biomarkers in NSCLC and their reported associations with ICI outcomes.

**Table 1:** Selected chemokine-related biomarkers in NSCLC and associations with immunotherapy outcomes.

Chemokine or immune marker	Association with immunotherapy response in NSCLC
CXCL8 (IL-8) – circulating (serum or plasma)	High baseline IL-8 correlates with poor response to ICIs and shorter survival. IL-8 recruits neutrophils and MDSCs, fostering an immunosuppressive, pro-angiogenic TME (20). In a meta-analysis of >1,500 patients, high IL-8 was associated with ~2.3-fold higher mortality risk under ICI therapy. Clinical trials are testing IL-8 blockade (e.g. anti-IL-8 antibody + nivolumab) to improve ICI outcomes (21).
CXCL9, CXCL10 – IFN-γ–inducible Th1 chemokines (tumor)	High intratumoral CXCL9/CXCL10 indicates a “T cell–inflamed” TME and is associated with better ICI response. Elevated CXCL9 and CXCL10 in pretreatment tumors correlate with greater CD8+ T-cell infiltration and improved responses to PD-1 blockade (22). Persistently high levels on-treatment, however, might reflect compensatory immune feedback and have been linked to resistance in some contexts (23).
CXCL13 – B cell/T-associated chemokine (tumor or blood)	High baseline CXCL13 is associated with favorable ICI outcomes. Linked to presence of TLS in tumors. NSCLC patients with higher CXCL13 levels showed improved response and survival on ICIs (24).
CCL5–CCR5 axis (tumor)	The CCL5-CCR5 axis exhibits significant context-dependency, frequently leaning towards a pro-tumorigenic role. While CCL5 is capable of recruiting CD8+ T cells, it also facilitates the influx of regulatory T cells (Tregs) and immunosuppressive monocytes. Notably, in KRAS-mutant lung adenocarcinoma, tumor-derived CCL5 has been shown to drive Treg recruitment, thereby fostering resistance to ICIs. Consequently, although CCR5 antagonism is being explored as a therapeutic strategy, its clinical application is complicated by the pleiotropic and context-specific effects of this signaling pathway (25).
CCL2, CCL7 – CCR2 ligands (tumor)	High CCL2/CCL7 indicates an immunosuppressive TME and poorer ICI outcomes. These chemokines recruit CCR2+ monocytes that become suppressive TAMs. Elevated CCL2/CCR2 activity is linked to T-cell exclusion and primary resistance. CCR2 inhibitors have shown synergy with ICIs in preclinical models by reducing TAMs/MDSCs (26).
CCL17, CCL22 – CCR4 ligands (tumor)	Associated with Treg-rich “cold” TMEs and reduced ICI efficacy. Attract CCR4+ Tregs that dampen anti-tumor immunity. High pre-treatment levels of circulating CCR4+ (and CCR8+) Tregs predict shorter PFS/OS with ICIs, suggesting these chemokines contribute to ICI resistance (27).
CXCR4+ – SDF-1 (CXCL12) receptor (tumor)	While the CXCL12-CXCR4 axis is traditionally recognized as a hallmark of aggressive, metastatic phenotypes and immune-excluded microenvironments, extreme overexpression of CXCR4 has been linked to paradoxical T-cell infiltration and sporadic responses to ICI therapy. High CXCR4 drives metastasis and is linked to worse survival in NSCLC. Trials are testing CXCR4 inhibitors + ICIs to improve T-cell infiltration of tumors (28).
CX3CR1 CD8 T cells (blood)	Emerging positive biomarker of ICI efficacy. CX3CR1 marks effector CD8 T cells. In a study, responders to PD-1 blockade showed an early expansion of circulating CX3CR1+ CD8 T cells. Such dynamic changes are being studied as a real-time indicator of response (e.g. trial monitoring peripheral CX3CR1+ T cells) (29).
CCR4 or CCR8 T cells (blood)	High levels of these mostly regulatory T cell subsets predict poor outcomes. Two independent analyses found NSCLC patients with elevated circulating CCR4+ or CCR8+ T regs pre-treatment had significantly shorter progression-free and overall survival on ICIs, consistent with a systemic immunosuppressive state that blunts immunotherapy efficacy (30).

TME, tumor microenvironment; ICI, immune checkpoint inhibitor; MDSC, myeloid-derived suppressor cell; TLS, tertiary lymphoid structure; T, T follicular helper cell; TAM, tumor-associated macrophage; PFS, progression-free survival; OS, overall survival; PD-1, programmed cell death-1; PD-L1, programmed death-ligand 1.

Generally, T cell–recruiting (Th1-type) chemokines are linked to favorable responses, whereas myeloid- and Treg-recruiting chemokines are linked to resistance (immune exclusion). For example, high intratumoral levels of CXCR3-binding CXC chemokines induced by interferon-γ (notably CXCL9 and CXCL10) are considered hallmarks of an “inflamed,” T-cell-infiltrated tumor phenotype, and elevated baseline expression of these chemokines is generally thought to favor a more durable response to PD-1 blockade by reflecting a pre-existing antitumor immune response in the TME (22). This relationship may not be static, however: in a large NSCLC cohort, acquired resistance following an initial response to PD-(L)1 blockade was characterized by persistent, rather than diminished, interferon-γ pathway activity, suggesting that sustained chemokine signaling can also accompany eventual treatment failure (23). These chemokine-centered observations have, in turn, been incorporated into broader multi-omic frameworks for predicting clinical endpoints after immunotherapy in NSCLC (31). Likewise, CXCL13, a B-cell-attracting chemokine involved in tertiary lymphoid structure (TLS) formation, has emerged as a favorable biomarker: in a cohort of patients with NSCLC, a higher intratumoral density of CXCL13-positive cells predicted response to anti–PD-1/PD-L1 therapy (24). In a complementary mouse study, the administration of CXCL13 increased intratumoral T-cell infiltration and improved PD-1 blockade efficacy, suggesting a causal role for this chemokine in potentiating anti-tumor immunity (32). These interferon-inducible Th1-type chemokines (CXCL9, CXCL10, and CXCL11) and TLS-associated chemokines (CXCL13) are often enriched in “hot” tumors, which generally respond better to ICIs (33).

In contrast, immunosuppressive and myeloid cell–recruiting chemokines tend to mark “cold” or excluded TMEs and are associated with ICI resistance (34). For instance, CXCL8 (IL-8), a potent neutrophil and myeloid-derived suppressor cell (MDSC) chemoattractant, can create a proangiogenic, immunosuppressive microenvironment. High tumor or plasma IL-8 levels correlate with increased neutrophil infiltration, elevated MDSCs, abnormal vasculature, and exclusion of cytotoxic T cells (35). Clinically, elevated IL-8 levels have been consistently associated with poor responses to ICIs and shorter survival in NSCLC and other cancers (36). A 2023 meta-analysis of >3,000 patients confirmed that high circulating IL-8 levels portend significantly worse overall survival in cancer patients treated with ICIs (36). These findings have motivated clinical trials of IL-8 pathway inhibition. BMS-986253, a monoclonal antibody against IL-8, is being evaluated in combination with nivolumab, with or without a CCR2/CCR5 antagonist, in a neoadjuvant trial enrolling patients with resectable NSCLC or hepatocellular carcinoma, with major pathologic response as the primary endpoint (37). BMS-986253 has also been tested in the advanced-disease setting, combined with nivolumab with or without ipilimumab, in a phase I/II basket trial across several tumor types; however, a randomized analysis of this combination in patients with advanced melanoma resistant to prior PD-(L)1 blockade showed no improvement in response rate or progression-free survival with the addition of anti-IL-8 to nivolumab plus ipilimumab, underscoring that the encouraging preclinical rationale for IL-8 blockade has not yet translated into a clear clinical benefit when combined with ICIs (38). Another strategy is blocking the receptors for CXCL8 (CXCR1/2); preclinical lung cancer models have shown that CXCR2 inhibition can reduce neutrophil recruitment and reprogram them to a less immunosuppressive state, thereby improving T-cell infiltration and enhancing antitumor effects when combined with ICIs (39).

Other tumor-derived chemokines that recruit regulatory or suppressive immune cells have also been linked to poor ICI outcomes. CCL2 (MCP-1) and CCL7, acting on CCR2, drive the accumulation of monocytes that differentiate into tumor-associated macrophages (TAMs), often polarized to an immunosuppressive “M2” phenotype, which can inhibit T cells and promote tumor progression (40). High CCL2/CCR2 activity in NSCLC is associated with increased TAM infiltration and poor prognosis (41). In preclinical models, blocking CCL2–CCR2 reduces MDSC and TAM accumulation, unleashing CD8+ T-cell activity, and synergizing with PD-1 blockade (42). Similarly, CCL17 and CCL22 (from tumor or stromal cells) engage CCR4 to recruit regulatory T cells (Tregs) into the TME; these chemokines are often elevated in Treg-rich lung tumors (43). High baseline frequencies of circulating CCR4+ or CCR8+ T cells (markers of Tregs) have been associated with significantly shorter progression-free and overall survival in patients with NSCLC treated with ICIs, presumably reflecting a systemic immunosuppressive milieu (44). The CCL5–CCR5 axis is more complex: CCL5 can attract CD8+ T cells (potentially beneficial), but also Tregs and pro-tumor macrophages, depending on the context (45). In mutant KRAS-driven lung tumors, cancer cell production of CCL5 is linked to increased Treg recruitment and worse outcomes (25). Conversely, some data suggest that CCL5 may contribute to immunogenic T-cell trafficking in certain settings. This context-dependent effect underscores that the net impact of a given chemokine depends on the balance of the immune cell subsets it recruits. **Table 1** includes CCL5 and other chemokines, where relevant.

Finally, chemokine pathways involved in metastasis and tissue niche formation can indirectly affect the response to immunotherapy. For example, CXCR4, the receptor for CXCL12 (SDF-1), is widely expressed in cancers and is classically associated with metastasis and poor prognosis in NSCLC (46). High tumor CXCR4 expression drives metastatic spread and is linked to aggressive disease and shorter survival (46). Paradoxically, some analyses indicate that extremely high CXCR4-expressing NSCLC tumors harbor more T-cell infiltration and may respond better to PD-1 blockade, possibly because these tumors induce certain chemokines (for example, CXCL10 and CXCL11) that attract immune cells, or because they elicit strong counter-regulatory immune responses that become susceptible to checkpoint inhibition (47). This finding has prompted trials combining CXCR4 antagonists (such as plerixafor or the peptide LY2510924) with ICIs to improve outcomes by overcoming stromal barriers to T-cell infiltration (28). Another intriguing biomarker is the presence of CX3CR1+ CD8+ effector T cells in the peripheral blood. CX3CR1 (fractalkine receptor) is associated with cytotoxic differentiation. In an exploratory study, NSCLC patients who responded to PD-1 inhibitors showed an increased proportion of circulating CX3CR1+ CD8 T cells shortly after therapy initiation compared to non-responders (29). This dynamic blood-based metric (indicative of mobilized effector T cells) is being explored prospectively as a real-time indicator of ICI efficacy, although dedicated trials formally validating this specific application in NSCLC are, to our knowledge, still lacking. Additionally, high baseline levels of certain chemokine-receptor–expressing T-cell subsets in the blood have shown prognostic value; for example, two independent analyses found that NSCLC patients with elevated pre-treatment frequencies of CCR4+ or CCR8+ T cells (predominantly Tregs) had significantly shorter progression-free and overall survival on ICIs (44).

In summary, chemokine profiles serve as important immune biomarkers that reflect the state of the tumor–immune interface. Pro-inflammatory (Th1) chemokine signatures denote a T cell–inflamed TME and are generally associated with better ICI responsiveness, whereas immunosuppressive chemokines denote “cold” tumors predisposed to primary resistance (48). This has spurred the development of chemokine-based composite biomarkers. For instance, one study defined a 7-chemokine gene signature that stratified patients with NSCLC by prognosis and correlated with immune cell infiltration patterns (49). Another genomic analysis in lung adenocarcinoma identified a five-marker mRNA signature, combining four chemokine ligands (CXCL2, CXCL13, CCL26, and CCL20) with one chemokine receptor (CX3CR1) rather than five chemokines proper, that was an independent prognostic factor and also associated with immunotherapy response, with high signature scores correlating with T-cell–inflamed, high-TMB tumors that responded better to ICIs (50). Efforts are underway to validate such multi-gene chemokine signatures in clinical assays. Ultimately, measuring chemokine and cytokine levels (in tumor tissue or blood) in conjunction with other immune markers could significantly enhance our ability to predict which patients with NSCLC will benefit from immunotherapy.

It is important to note, however, that the predictive value of chemokines is not universal and varies substantially across several biological and analytical dimensions. First, histologic subtype matters: lung adenocarcinomas and squamous cell carcinomas differ in their baseline immune infiltration and chemokine receptor expression profiles, so signatures validated in one histology may not translate directly to the other. Second, the sampling compartment introduces meaningful differences; tumoral and circulating levels of the same chemokine often behave discordantly, and a biomarker informative in tumor tissue may lack predictive value in plasma, or vice versa. Third, treatment line and combination regimen influence chemokine dynamics: for example, chemotherapy administered alongside ICIs can transiently alter CXCL9/CXCL10 gradients and neutrophil–CXCL8 interactions in ways that differ from ICI monotherapy. Fourth, the analytic platform (multiplex immunoassay, RNA-based sequencing, or mass spectrometry proteomics) affects quantification thresholds and inter-laboratory reproducibility. Fifth, as discussed above for CCL5 and CXCR4, individual chemokines can exert opposing net effects depending on the balance of cell types they recruit in a given microenvironmental context. These sources of variability imply that chemokine biomarkers should ideally be evaluated in the specific clinical context in which they are intended to be used, rather than extrapolated wholesale from studies conducted under different histologic, treatment, or platform conditions. Multi-omic validation across independent cohorts representing diverse patient populations and treatment settings is therefore essential before any single chemokine signature can be considered ready for clinical implementation.

## 4 Multi-omic biomarkers and integrated signatures for immunotherapy response

**Figure 1:**
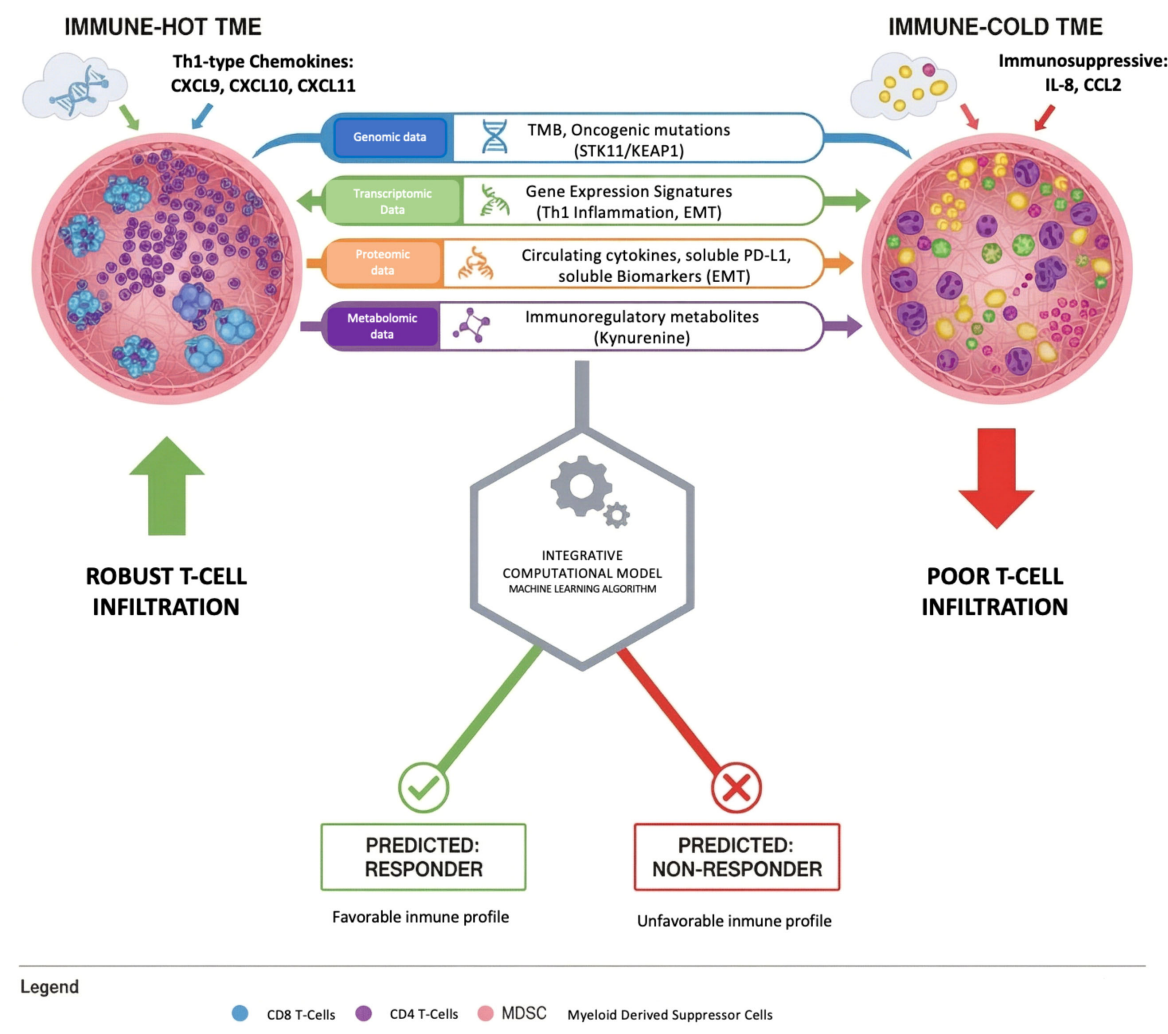
Integrative framework combining chemokine signatures and multi-omic biomarkers to predict immunotherapy response in non-small cell lung cancer (NSCLC). Schematic representation of an integrative biomarker model illustrating how chemokine-driven tumor microenvironment (TME) states are combined with multi-omic data to estimate the likelihood of response to immune checkpoint inhibitors. Two contrasting TME phenotypes are shown: an immune-inflamed (“hot”) TME characterized by Th1-type chemokines (e.g., CXCL9, CXCL10, CXCL13), robust CD8^+^ T-cell infiltration, and tertiary lymphoid structure formation; and an immune-suppressed (“cold”) TME enriched in immunosuppressive chemokines (e.g., IL-8, CCL2), associated with recruitment of neutrophils, myeloid-derived suppressor cells, or regulatory T cells and limited T-cell infiltration. These chemokine-defined immune contexts are integrated with complementary multi-omic biomarkers derived from tumor tissue and peripheral blood, including genomic features (e.g., tumor mutational burden and oncogenic alterations such as *STK11* or *KEAP1*), transcriptomic signatures of immune activation or epithelial–mesenchymal transition, circulating proteomic markers (e.g., cytokines and soluble PD-L1), and immunoregulatory metabolites. Integration of these data layers through a centralized computational or machine-learning model generates a composite immunotherapy response classification, stratifying patients into predicted responders or non-responders. This framework highlights how multidimensional biomarker integration can capture the complexity of tumor–immune interactions and support improved patient stratification in NSCLC.

Before reviewing the specific multi-omic approaches, it is useful to situate the major biomarker classes in relation to each other (**Table 2**). PD-L1 immunohistochemistry and TMB by next-generation sequencing are the only companion-diagnostic tools approved for first-line NSCLC immunotherapy, but both carry well-documented limitations in sensitivity and specificity. Chemokine-based transcriptomic signatures (such as the Th1-type CXCL9/CXCL10 signature or the seven-gene chemokine score) provide an indirect readout of immune-cell trafficking and TME polarization; they have been evaluated primarily in retrospective or translational cohorts and lack prospective clinical trial validation, though they consistently add predictive information on top of PD-L1 alone. Broader transcriptomic immune signatures (e.g., the T-cell-inflamed GEP) are at an intermediate stage, having been analytically validated alongside randomized trial data, but are not yet approved as stand-alone companion diagnostics. Blood-based circulating proteomic markers (serum IL-8, multiplex cytokine panels, soluble PD-L1) and circulating immune-cell phenotypes (CX3CR1+ CD8+ T cells, CCR4+ Tregs) offer the advantage of non-invasive sampling and serial monitoring, but most remain at the discovery or early retrospective-validation stage, with limited data from prospective trials. Machine-learning-based composite models, which integrate genomic, transcriptomic, and proteomic features, consistently outperform single-analyte tests in retrospective series and some external validation cohorts; however, they face significant barriers to clinical adoption, including the need for prospective validation, assay standardization, interpretability, and cost. Recognizing these distinctions is important when interpreting the evidence reviewed below. While single factors such as PD-L1 or cytokines provide limited predictive power, integrating multiple biomarker modalities (“multi-omics”) offers a more comprehensive view of tumor and host immune biology (51). The conceptual framework of integrating chemokine signatures with genomic, transcriptomic, proteomic, and metabolomic biomarkers to stratify immunotherapy responders and non (**Figure 1**).

**Table 2:** Examples of multi-omic biomarkers for immunotherapy and their clinical relevance in NSCLC.

Biomarker or signature	Clinical relevance and predictive utility
Tumor PD-L1 expression (IHC)	Most established biomarker. High tumor PD-L1 expression (≥50% TPS) is associated with higher response rates and longer progression-free survival (PFS) to PD-1/PD-L1 inhibitors. Used worldwide to select first-line pembrolizumab therapy in NSCLC. Limitations: marked spatial and temporal heterogeneity and imperfect specificity (many PD-L1–high patients do not respond, while some PD-L1–negative patients derive benefit).
Tumor mutational burden (TMB)	Emerging approved biomarker. High TMB correlates with increased neoantigen load and improved ICI response in multiple studies. FDA-approved in 2020 for pembrolizumab in TMB-high (≥10 mut/Mb) solid tumors. In NSCLC, TMB-high status enriches for responders but is not absolute; co-mutations such as STK11 or KEAP1 may negate benefit despite high TMB. Challenges: tissue or blood DNA requirements, inter-assay variability, and debated optimal cutoffs.
DNA repair defects and mutational signatures (NGS)	Promising genomic markers. Certain genomic contexts (e.g. POLE mutations or high microsatellite instability) generate hypermutated tumors with exceptional ICI responses, though these are rare in NSCLC (~1% or less). Conversely, EGFR or ALK driver alterations are associated with primary resistance to ICIs; these patients benefit more from TKIs and ICIs are not first-line therapy.
T-cell–inflamed Gene Expression Profile (GEP) (Tumor RNA)	Research-use mRNA signature. Quantifies IFN-γ–responsive, Th1-associated genes (e.g. CXCL9/10, IDO1, PRF1, HLA-DR genes). A high GEP score reflects a pre-existing antitumor immune response (“inflamed” TME) and correlates with improved ICI outcomes in NSCLC, independently of PD-L1. Not yet routine; under evaluation in clinical trials and retrospective cohorts.
Other tumor mRNA signatures (RNA-seq)	Exploratory. Multiple gene-expression programs have been proposed. An “immune-exhaustion” signature (high checkpoint expression with metabolic/stromal/EMT genes) identifies tumors with primary resistance. 7-gene and 5-gene chemokine signatures show prognostic value and may predict ICI benefit. Single-cell and spatial transcriptomics delineate immune niches (e.g. TLS-rich vs neutrophil-rich regions) linked to response or resistance. Not yet clinical tools, but key for biomarker discovery.
Tumor-infiltrating lymphocytes (TILs) and TLS	Qualitative immune-contexture markers. High baseline intratumoral CD8^+^ T-cell density is generally associated with better ICI outcomes, whereas low TILs or immune-excluded phenotypes indicate resistance. Tertiary lymphoid structures (TLS) are emerging favorable biomarkers, often linked to CXCL13 expression and improved responses. Sometimes assessable by routine histology/IHC; being formally quantified in trials.
Peripheral blood cell counts	Readily available but nonspecific. High neutrophil-to-lymphocyte ratio (NLR) or derived NLR associate with poorer ICI outcomes, reflecting systemic inflammation. The lung immune prognostic index (LIPI) (high NLR + high LDH) identifies patients less likely to respond. Easy to obtain but influenced by comorbidities; not used for selection.
Circulating cytokines and proteins	Non-invasive immune monitoring. Elevated immunosuppressive factors (IL-6, IL-8, VEGF) associate with poor ICI outcomes; high IL-8 strongly correlates with reduced response and survival. High soluble PD-L1 levels in plasma have also been linked to inferior outcomes. Not yet clinical; multiplex cytokine panels (e.g. 5- or 8-protein signatures) are under development.
Circulating tumor DNA (ctDNA) and mutation tracking	Liquid biopsy markers. High baseline bTMB has been associated with ICI benefit in retrospective studies, though prospective validation has yielded mixed results. On-treatment ctDNA clearance correlates with radiographic response and longer survival, making ctDNA dynamics a promising early predictor. Clinically available for genotyping; immunotherapy monitoring remains investigational.
Multi-parametric composite scores	Cutting-edge integration. Models combining clinical features with tumor and immune biomarkers (e.g. IMPRES, IO scores, machine-learning models) show promising predictive performance. Highly promising but require prospective validation; none yet routine.

IHC, immunochemistry; TPS, tumor proportion score; NLR, neutrophil-to-lymphocyte ratio; LDH, lactate dehydrogenase; LIPI, Lung Immune Prognostic Index; bTMB, blood tumor mutational burden; VEGF, vascular endothelial growth factor; AUC, area under ROC curve.

Modern high-throughput techniques enable the parallel profiling of the cancer genome, epigenome, transcriptome, proteome, and even the microbiome, generating a wealth of data that can be mined for patterns predicting therapy response (52). In NSCLC, several multi-omics–based predictive approaches have been developed in recent years.

- Genomic and epigenomic markers: beyond TMB, specific genetic alterations have been linked to ICI outcomes. For example, mutations in *STK11 (LKB1)* and *KEAP1* (frequent in NSCLC, especially lung adenocarcinoma) are associated with poor responses to PD-1/PD-L1 inhibitors, even in tumors with high TMB (53). *STK11* or *KEAP1* mutant tumors often lack T-cell infiltration and have an immunosuppressive environment, reducing ICI efficacy. Conversely, certain genomic features may confer sensitivity, such as tumors with DNA replication repair deficiencies (for example, POLE mutations or high microsatellite instability) exhibit very high neoantigen loads and have shown improved responses to ICIs (54). Similarly, TP53 co-mutations with *KRAS* have been associated with an inflamed TME and better outcomes, in contrast to *KRAS* plus *STK11/KEAP1* co-mutations, which yield “cold” tumors (55). At the epigenetic level, global DNA methylation patterns and chromatin modifications may influence immune infiltration; for instance, tumors with lower methylation burden or “unshielded” chromatin at immune gene loci could be more visible to the immune system (56), although robust epigenetic predictors of ICI response are still under investigation. Large genomic studies have also revealed mutation-based signatures: one study derived a 27-gene “immune resistance” mutation signature that predicted poor response and survival after immunotherapy, validating it in multiple cohorts (57). These genetic and epigenetic markers often need to be interpreted in combination with other features to achieve clinically useful predictive accuracies.
- Transcriptomic signatures (gene expression profiles or GEP): gene expression profiling captures the functional state of tumor and immune cells. A prominent example is the “T-cell–inflamed GEP”, a score reflecting the expression of multiple T_h1_ -associated immune genes (for example, CD8A, IFNγ, CXCL9, LAG3, etc.) (58). A high T-cell–inflamed GEP indicates a pre-existing antitumor immune response and has been associated with improved response to PD-1 blockade in NSCLC and other cancers, independent of PD-L1 status (11). For instance, in one study, patients with NSCLC with a high pre-treatment T_h1_ -oriented GEP had significantly better outcomes with pembrolizumab, even after adjusting for PD-L1 expression (59). In contrast, gene signatures reflecting immunosuppression or epithelial-mesenchymal transition (EMT) were correlated with resistance. Tumors with high expression of EMT-related genes and angiogenesis factors often manifest an “immune-excluded” phenotype (T cells present at the margins but unable to penetrate the tumor), resulting in poor ICI responses (60). For example, an EMT/stroma-related mRNA signature was associated with primary resistance to ICIs, likely due to a non-inflamed, T cell–excluded TME (61). Conversely, high expression of antigen presentation machinery genes, cytotoxic effector molecules, and chemokines such as CXCL9 and CXCL13 (essentially an “inflamed” gene profile) usually denotes an immune-reactive tumor likely to respond well (62). Unsupervised machine learning has been used to define holistic transcriptional subtypes of NSCLC with distinct immunotherapy outcomes (63). For instance, a recent analysis identified a subset of NSCLC tumors characterized by the concurrent upregulation of immune checkpoints, chemokines, and metabolic pathways; these tumors had particularly poor responses to ICIs, suggesting the presence of multiple coordinated resistance mechanisms at play (64). Emerging techniques, such as single-cell RNA sequencing and spatial transcriptomics, provide new insights by resolving the spatial and cellular context of gene expression patterns. A 2024 study by Aung et al. used spatial multi-omics (combining single-cell protein imaging with spatially resolved transcriptomics) to define distinct immune microenvironment “spatial signatures” in NSCLC (65). Tumors enriched in TLS and IFN-activated gene regions had superior ICI responses, whereas tumors with spatial niches dominated by neutrophils, blood vessels, and EMT-related gene expression were associated with primary resistance (65). These high-dimensional approaches underscore that combined patterns of gene expression, rather than single genes, are key to predicting the benefits of immunotherapy.
- Proteomic and metabolomic markers: blood-based proteomics has revealed circulating protein signatures with predictive power. Instead of looking at one cytokine in isolation, investigators have identified panels of cytokines and soluble factors related to immune activation or suppression. For example, one study found that an 8-protein serum signature (including inflammatory cytokines such as IL-6, IL-1β, and CXCL8, among others) could distinguish responders from non-responders to PD-1 inhibitors in NSCLC with >80% accuracy, outperforming any single analyte (66). Another group described a 5-protein plasma biomarker panel that added predictive value to PD-L1 status (67). These multi-analyte assays are being advanced to prospective trials; one such proteomic assay (the “INSPIRE” score or PROphet score) combines levels of multiple cytokines and angiogenic factors to predict ICI benefit, and is being validated alongside PD-L1 in clinical studies (68, 69). Beyond cytokines, the metabolic state of the patient and tumor can influence immunotherapy response. Metabolomic profiling has shown that certain metabolites may modulate immunity: e.g., high serum kynurenine (a tryptophan metabolite produced by indoleamine 2,3-dioxygenase, IDO) is immunosuppressive and has been linked to poor ICI outcomes (70). Other work suggests that metabolomic signatures reflecting an active tricarboxylic acid (TCA) cycle or specific gut microbiota–derived short-chain fatty acids might correlate with better responses (71). These areas remain under active investigation. Additionally, autoantibodies against certain tumor antigens have been proposed as biomarkers; for instance, baseline presence of anti–NY-ESO-1 or other tumor antigen antibodies might indicate an immunologically primed host, potentially correlating with improved ICI efficacy (72). Though intriguing, such findings need validation.
- Composite and integrated multi- modal signatures: the most powerful predictive models will likely arise from combining multiple types of data. Recently, studies have demonstrated that integrative approaches (using clinical factors plus combinations of genomic, transcriptomic, and proteomic features) achieve more accurate prediction of ICI outcomes than any single biomarker alone (73). For example, one group integrated tumor genomics (TMB and neoantigen profiles), a 18-gene T-cell–inflamed expression signature, and select serum proteins to create a composite immunotherapy response predictor; this model achieved a high area under the receiver-operating characteristic curve (AUC) in distinguishing responders from non-responders, whereas models based on TMB or PD-L1 alone were less discriminative (74). Another recent study by Chen et al. used machine learning to integrate multi-omics data (genomic mutations, gene expression, and clinicopathologic features) in a random survival forest model. The resulting risk score separated NSCLC patients by likelihood of response to ICIs more effectively than PD-L1 or TMB alone (with an AUC >0.70 in validation cohorts) (75). Notably, patients with low integrated risk scores had “hot” immune microenvironments (e.g. high CD8+ T-cell infiltration and low immunosuppressive cell levels) and were much more likely to benefit from ICIs (75). The model’s utility was confirmed across multiple independent cohorts including a landmark trial (IMvigor210 in urothelial cancer) (74), underscoring its potential generalizability. Other groups have developed multi-omics signatures using advanced algorithms: for example, Zhang et al. constructed a 23-gene combined risk signature (encompassing genes related to cell death pathways and organelle stress) that predicted outcomes on ICIs and identified “cold” tumors with immunosuppressive TMEs, suggesting those patients might need combination therapies beyond PD-1 monotherapy (76). Deep learning methods are also being applied to “pan-omic” data; Wang J et al, showed that a neural network could integrate genomic, transcriptomic, and histopathological image features to predict anti–PD-1 response with improved accuracy over conventional models (77). These strategies are not limited to tumor tissue: notably, researchers are exploring liquid-biopsy–based multi-omic predictors, for example, combining circulating tumor DNA (ctDNA) genomic alterations with plasma cytokine levels and peripheral immune cell phenotypes to create a comprehensive “immune response score.” Early results suggest that such liquid-based multi-parametric scores can stratify responders and non-responders without an invasive biopsy (78). In the coming years, it is conceivable that oncologists will use integrated diagnostic tests analyzing multiple layers of tumor and immune data simultaneously to guide immunotherapy decisions, rather than relying on any single biomarker. It must be emphasized, however, that the majority of the composite and machine-learning models described here remain at the retrospective-exploratory or single-cohort-validation stage. Very few have undergone formal prospective evaluation as primary endpoints in clinical trials, and none is currently approved as a companion diagnostic for NSCLC immunotherapy. Key methodological concerns, overfitting to training datasets, assay non-standardization, model opacity, and poor performance on external populations that differ in ethnicity or tumor subtype, have been documented repeatedly and should caution against premature clinical adoption. Translating these models into routine practice will require, at a minimum, pre-specified biomarker analyses in prospective randomized trials, standardized analytical platforms, and independent regulatory-grade validation (73).

## 5 Challenges in biomarker development and implementation

Despite the promise of these emerging biomarkers, significant challenges must be addressed before they can be widely adopted in clinical practice. Biological complexity and heterogeneity remain major hurdles. The cancer immune interaction is influenced by numerous dynamic factors (transient inflammation, infections, or concomitant therapies can alter circulating cytokine levels and immune cell distributions), potentially confounding their interpretation as cancer-specific biomarkers. Within the tumor, immune infiltrates and chemokine gradients can be patchy and evolve over time or with treatment, so single-site biopsies may not capture the full picture of the TME (79). Multi-region profiling or circulating assays might partly overcome this issue, but introduce new complexity.

Another challenge is assay standardization and validation. Multi-omic approaches produce enormous datasets, raising the risk of overfitting and false discoveries if not carefully validated. Rigorous standardization of assays and analytical pipelines is required. For example, different DNA sequencing platforms may report different TMB values, and gene expression results can vary with RNA-seq vs. NanoString vs. microarray methods (80, 81). Even PD-L1 IHC assays from different vendors were initially not standardized, prompting initiatives like the Blueprint Project to harmonize scoring (82). Similar efforts are needed for complex biomarkers: establishing reference standards for TMB calculation (83, 84), uniform protocols for multiplex immunofluorescence and digital pathology quantification of immune cells, and guidelines for reporting multi-gene signatures will be crucial. Prospective validation in clinical trials is essential to demonstrate that using a novel biomarker to guide therapy actually improves patient outcomes (e.g. by enriching responders or sparing non-responders from toxicity). Several such trials are ongoing or planned, including studies evaluating composite biomarkers in the context of first-line NSCLC immunotherapy.

From a clinical standpoint, an additional challenge is integration into decision-making. Oncologists are accustomed to simple, binary biomarkers (e.g. “PD-L1 high vs low”) to guide treatment. In contrast, many multi-omic predictors yield a continuous risk score or multidimensional output that may not have an obvious cutoff. Defining clinically actionable thresholds for such scores is non-trivial (85, 86). For instance, what constitutes a “positive” chemokine signature or a high immune composite score? Calibrating these cut-points for routine use will require large datasets and prospective analysis. Moreover, complex models must be made transparent and interpretable to gain physician trust, especially if driven by machine learning (“black box”) algorithms. This has spurred interest in explainable AI methods to ensure that model predictions can be understood in terms of biological features (87, 88).

Practical considerations and access also pose significant barriers. Some advanced biomarker tests (e.g. whole-exome sequencing for TMB, or comprehensive multiplex immunoprofiling) are expensive and not widely available outside academic centers (89, 90). In resource constrained settings, even basic PD-L1 testing or NGS may be hard to implement. Ensuring equitable access to predictive biomarker testing will require infrastructure investment and possibly less costly surrogate assays (such as blood-based tests). The turnaround time for complex multi-omic assays is another concern; treatment decisions in advanced cancer often must be made quickly, so lengthy or logistically complicated tests may not be practical. Simplified assays or rapid sequencing technologies could help address this issue. Regulatory and reimbursement frameworks will also influence implementation. For example, regulatory frameworks analogous to those established by the U.S. Food and Drug Administration for tumor-agnostic biomarker approvals (such as the tissue-independent approval of pembrolizumab for TMB-high solid tumors), will be required for the clinical deployment of multi-omic composite scores, as these tools must demonstrate analytical validity, clinical validity, and clinical utility across diverse tumor types and patient populations before they can be incorporated into routine oncological decision-making (91).

Finally, the generalizability of predictive models is an ongoing concern. Many promising biomarkers emerge from retrospective analyses of clinical trial populations that may not reflect “real-world” patient diversity (92, 93). For instance, machine learning models trained on one dataset can perform poorly when applied to external datasets that differ in patient demographics, tumor subtypes, or sample processing methods (92, 94). Large collaborative efforts, such as the Friends of Cancer Research Tumor Mutational Burden Harmonization Project, demonstrate the feasibility of multi−institutional data pooling and analytic standardization, enabling improved comparability across platforms and enhancing the robustness of downstream predictive model (80). Similarly, the critical importance of testing predictive signatures across multiple independent cohorts is well recognized, as repeated external validation is necessary to assess generalizability, quantify performance heterogeneity, and support clinical implementation (95). Public–private consortia are beginning to amass the large, harmonized multi−omic datasets needed to refine and validate complex biomarkers at scale. For example, the Tumor nEoantigen SeLection Alliance (TESLA) has evaluated multi−parameter predictors of immunotherapy response across international cohorts to identify reproducible signals, while complementary NCI−led efforts such as the CIMAC–CIDC network enable standardized, cross−trial multi−omic analyses of ICI response (96, 97). .

## 6 Future directions

**Figure 2:**
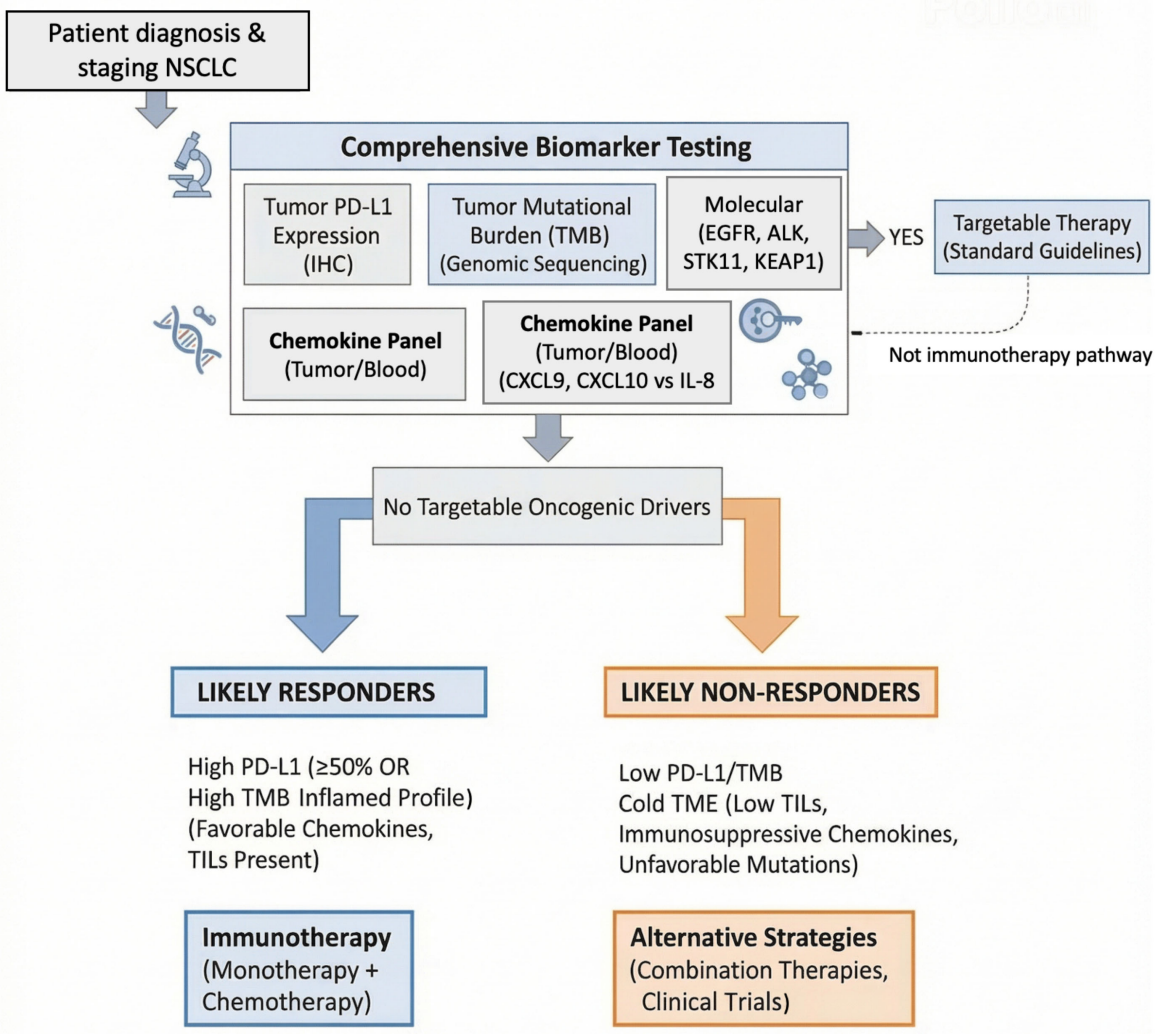
Biomarker-driven decision framework for patient selection in immunotherapy for non-small cell lung cancer (NSCLC). Proposed biomarker-based clinical flowchart illustrating a stepwise approach to patient stratification for immune checkpoint inhibitor therapy in NSCLC. Following diagnosis and staging, patients undergo comprehensive biomarker assessment, including tumor PD-L1 expression, tumor mutational burden, oncogenic driver alterations, and chemokine profiling from tumor tissue or peripheral blood. Detection of actionable driver mutations directs patients toward guideline-recommended targeted therapies outside the immunotherapy pathway. For patients without targetable oncogenic drivers, integrated evaluation of tumor-intrinsic biomarkers and immune contexture informs treatment selection. Tumors characterized by high PD-L1 expression and/or elevated tumor mutational burden in the context of an immune-inflamed microenvironment—defined by favorable chemokine signatures and tumor-infiltrating lymphocytes—are classified as likely responders and considered candidates for immunotherapy, either alone or in combination with chemotherapy. Conversely, tumors with low PD-L1/TMB and an immune-suppressed microenvironment, marked by immunosuppressive chemokines and adverse molecular features, are classified as likely non-responders and may be better suited for alternative strategies, including combination regimens or enrollment in clinical trials. This framework emphasizes the translational integration of molecular and immune biomarkers to support personalized treatment decisions in NSCLC immunotherapy.

The future of biomarker-driven immunotherapy in NSCLC lies in continued advancements in multi-omic integration and deeper understanding of the TME. We anticipate several key developments in the coming years:

- Refinement of multi-modal predictive models: ongoing research will likely yield refined algorithms that combine clinical, pathological, and multi-omic features into user-friendly predictive tools. For example, nomograms incorporating inflammatory indices such as the lung immune prognostic index, transcriptomic signatures including chemokine expression, and standard clinical variables have demonstrated the potential to generate individualized risk scores for ICI response (48, 98, 99). The study by Zhao et al. exemplifies this approach: they constructed a multidimensional prognostic model integrating a radiomics score, an immune cell infiltration score, and clinical indices, which outperformed any single metric in predicting NSCLC patient outcomes on ICIs (100). As more data become available, models will be further optimized and validated. Importantly, success will require close collaboration between data scientists, immunologists, and clinicians to ensure that model outputs are both accurate and interpretable in a clinical context.
- Non- invasive biomarker development: there will be a strong push toward liquid biopsy and blood-based predictors. Analyzing circulating tumor DNA, exosomal RNA/proteins, and immune cell subsets in peripheral blood offers a real-time window into the evolving tumor-immune dynamics during therapy (48, 101, 102). For example, serial monitoring of ctDNA for emerging mutations or clearance of tumor-specific variants can provide early indication of response or resistance, preceding radiographic changes. Similarly, tracking changes in circulating T-cell populations (like the rise in CX3CR1+ CD8 T cells) or cytokine levels on therapy might alert clinicians to adjust treatment strategies earlier in the course. By integrating these with baseline tissue genomics and longitudinal imaging (e.g. radiomic analysis of CT scans), clinicians could have a cohesive view of each patient’s response trajectory.
- Targeting the tumor microenvironment: represents a promising translational extension of chemokine biomarker research. Beyond their predictive value, chemokine−associated pathways highlight actionable therapeutic opportunities, and ongoing studies are evaluating combinations of ICI with agents that modulate the tumor microenvironment. For CXCR4 antagonism specifically, the most direct clinical evidence to date comes from a phase 2 trial in metastatic pancreatic adenocarcinoma, where combining a CXCR4 antagonist with PD-1 inhibition recruited T cells into tumors but also immunosuppressive macrophages (103); whether this strategy, or its net benefit, translates to NSCLC has not yet been tested and should not be assumed. Other strategies under earlier-stage exploration include CCR4 inhibitors targeting regulatory T−cell trafficking and cytokine−directed approaches such as IL−8 or TGF−β inhibition (36, 104). Collectively, this approach reflects a shift toward biomarker−driven combination immunotherapy, guiding not only whether to administer an ICI, but also which complementary agents might rationally be co−administered to overcome context−specific resistance mechanisms, pending dedicated NSCLC trials.
- Computational pharmacology and drug repositioning: network-medicine and genome-scale computational pharmacology platforms represent an emerging, complementary strategy for nominating therapeutic agents based on the same multi-omic vulnerabilities discussed throughout this review. Comprehensive CRISPR-based gene-essentiality maps and genome-scale functional-module transformations have been developed to quantitatively link gene-dependency and drug-efficacy data across large panels of cancer cell lines, offering a scalable means of prioritizing candidate combination partners for specific molecular contexts (105, 106). Pharmacotranscriptomic screening platforms originally developed for natural-product and herbal-ingredient discovery have similarly been adapted to quantify how individual compounds remodel the tumor immune microenvironment and reprogram metabolism (107, 108). As a proof of concept with direct relevance to checkpoint blockade, a network-medicine-based drug-repositioning framework recently identified the antidepressant maprotiline as a repurposable agent that reduces PD-L1 expression by targeting the E3 ubiquitin ligase SPOP, with preclinical evidence of enhanced antitumor activity when combined with checkpoint blockade (109). None of these platforms has yet been validated specifically in NSCLC immunotherapy cohorts, but collectively they illustrate how computational pharmacology could, in principle, be integrated with the chemokine and multi-omic signatures reviewed here to nominate rational, biomarker-guided combination regimens for prospective testing.
- Personalized immunotherapy and adaptive algorithms: ultimately, the integration of chemokine signatures and multi-omics is bringing oncology closer to true precision immunotherapy. A future clinical paradigm may integrate at baseline, an NSCLC patient’s tumor biopsy is profiled for PD-L1, DNA mutations, RNA signatures (possibly via a targeted panel or whole transcriptome assay), while blood is tested for key cytokines and ctDNA, and even the gut microbiome is analyzed, collectively informing an AI-driven predictive algorithm that outputs a probability of response to various therapies. A biomarker-driven clinical decision flowchart integrating tumor genomics, immune biomarkers, and chemokine profiles for patient selection in NSCLC immunotherapy is proposed in **Figure 2**. High-probability patients might receive single-agent anti–PD-1, whereas low-probability patients might be triaged to alternate approaches (combination immunotherapy, addition of a novel immunomodulator, or even redirection to targeted therapy or chemotherapy). With iterative machine learning, such models can continuously improve. However, realizing this vision will require addressing the challenges described above: standardizing assays, sharing data across institutions, and carefully validating models in prospective trials to demonstrate improved patient outcomes.

## 7 Conclusions

In conclusion, the integration of chemokine signatures with multi−omic biomarkers holds substantial promise for advancing precision immunotherapy in NSCLC. Chemokine profiles offer critical insight into the immunological state of the tumor microenvironment, while multi−omic approaches capture the complex interplay of tumor−intrinsic and host−related factors that ultimately shape responses to ICI. Accumulating evidence indicates that no single biomarker is sufficient to account for this complexity; rather, a multidimensional framework is required to reflect the dynamic nature of tumor-immune interactions.

Continued research will be essential to refine these integrated predictive models, address challenges related to validation, standardization, and clinical implementation, and ensure that emerging advances are generalizable across diverse patient populations. As this field matures, biomarker−informed strategies are expected to move beyond predicting benefit from immune checkpoint inhibition alone, toward guiding rational combination approaches tailored to the tumor microenvironment. Ultimately, such advances will better equip clinicians to personalize immunotherapy for patients with NSCLC, matching each individual not only to the most appropriate immunotherapeutic agent, but also to the complementary strategies most likely to achieve durable tumor control, thereby improving outcomes while minimizing unnecessary toxicity in this challenging disease.
